# Insulin Secretion and Insulin Sensitivity Change in Different Stages of Adult-Onset Type 1 Diabetes: A Cross-Sectional Study

**DOI:** 10.3390/jcm14041109

**Published:** 2025-02-09

**Authors:** Tanja Milicic, Aleksandra Jotic, Katarina Lalic, Ljiljana Lukic, Marija Macesic, Jelena Stanarcic Gajovic, Milica Stoiljkovic, Mina Milovancevic, Djurdja Rafailovic, Aleksandra Bozovic, Nebojsa M. Lalic

**Affiliations:** 1 Clinic for Endocrinology, Diabetes and Metabolic Diseases, University Clinical Centre of Serbia, Doktora Subotica 13, 11000 Belgrade, Serbia; aleksandra.z.jotic@gmail.com (A.J.); katarina.s.lalic@gmail.com (K.L.); ljikson17@gmail.com (L.L.); macesicmarija@gmail.com (M.M.); stanarcicjelena@gmail.com (J.S.G.); mmstoiljkovic@yahoo.com (M.S.); milovancevicmina3@gmail.com (M.M.); djurdja.rafailovic@gmail.com (D.R.); alex91@hotmail.rs (A.B.); nebojsa.m.lalic@gmail.com (N.M.L.); 2 Faculty of Medicine, University of Belgrade, Doktora Subotica 8, 11000 Belgrade, Serbia

**Keywords:** recent-onset type 1 diabetes, pre-stage 3 type 1 diabetes, insulin secretion, insulin sensitivity, interleukin-17

## Abstract

**Background/Objectives**: Previous studies reported impairments in insulin secretion during different stages of type 1 diabetes (T1D), while data regarding insulin sensitivity and immunological changes are still controversial. We analyzed the following: (a) insulin secretion, (b) insulin sensitivity, and (c) pro-inflammatory interleukin-17 (IL-17) levels in peripheral blood in 17 healthy first-degree relatives in stage 1 (FDRs1) (GAD^+^, IA2^+^), 34 FDRs in stage 0 (FDRs0) (GAD^−^, IA2A^−^), 24 recent-onset T1D (R-T1D) patients in the insulin-requiring state (IRS), 10 in clinical remission (CR), and 18 healthy unrelated controls (HC). **Methods**: Insulin secretion was evaluated by an IVGTT and a glucagon stimulation test, expressed as a first-phase insulin response (FPIR) and a basal/stimulated C-peptide. Insulin sensitivity was tested by the euglycemic hyperinsulinemic clamp, expressed as an M value. **Results**: FDRs1 had a lower FPIR than FDRs0 (*p* < 0.05) and HC (*p* < 0.001) but higher than RT1D-IRS (*p* < 0.001) and RT1D-CR (*p* < 0.01). Moreover, FDRs1 had lower basal/stimulated C-peptide than FDRs0 (*p* < 0.01/*p* < 0.05) and HC (*p* < 0.001/*p* = 0.001) but higher levels than RT1D-IRS (*p* < 0.001/*p* < 0.001). However, the M value was similar among FDRs1, FDRs0, and HC (*p* = 1.0) but higher than RT1D-IRS (*p* < 0.001) and RT1D-CR (*p* < 0.01), while RT1D-IRS and RT1D-CR had lower M than HC (*p* < 0.001; *p* < 0.001; respectively). FDRs1 had higher IL-17 than FDRs0 (*p* < 0.001) and HC (*p* < 0.05). RT1D-IRS had higher IL-17 than FDRs0 (*p* < 0.001) and HC (*p* < 0.001), which was similar to RT1D-CR vs. FDRs0 (*p* < 0.001) and HC (*p* < 0.05). **Conclusions**: Early changes in pre-T1D might involve an initial decline of insulin secretion associated with a pro-inflammatory attack, which does not influence insulin sensitivity, whereas later, insulin sensitivity deterioration seems to be associated with the prominent reduction in insulin secretion.

## 1. Introduction

Type 1 diabetes (T1D) is an autoimmune disease based on autoimmune attacks, which can selectively destroy pancreatic β-cells years before clinical onset [1]. A previous study suggested that the period of prediabetes consists of different presymptomatic stages (0, 1, 2), which precede clinically manifested T1D (stage 3) [2]. Stage 0 refers to subjects with only genetic/familial risk for T1D without autoantibodies on pancreatic antigens, like first-degree relatives (FDRs) of patients with T1D who have multiple times higher risk levels for T1D than other people [3]. Next stage, stage 1, includes subjects with 1–2 autoantibodies on β-cell antigens and euglycemia, while in stage 2, subjects have 2 or more autoantibodies but with dysglycemias, and the highest risk is for stage 3 and development of overt T1D [2]. In addition, during the progression to overt T1D, there are differences between children and adults [4,5].

Although immunological markers have been extensively studied throughout the different stages of T1D [6], prediabetes’ metabolic course has not yet been elucidated. Previously, a substantial role of detriment in insulin secretion in prediabetes and at the beginning of manifested T1D was reported [7,8], mainly in the pediatric population. However, the data evaluating endogenous insulin secretion’s capacity in different stages of T1D in adults are still limited. Furthermore, the data concerning insulin sensitivity levels in previous stages continue to be questionable [9,10].

In addition, the onset of T1D is associated with the dominance of pro-inflammatory diabetogenic interleukin 17 (IL-17)-secreting Th17 cells [11,12]. In this context, it is shown that IL-17 is an important driver of destructive inflammation in T1D pathogenesis [13,14] in animal models, suggesting it may have a potential role in the initiation and progression of impairments in insulin secretion in humans pre-T1D.

However, the level of this proinflammatory cytokine and the investigation of metabolic and immunological/pro-inflammatory changes during preclinical adult T1D in FDRs and in recent-onset T1D (R-T1D) patients have not yet been elucidated.

In this context, this study aimed to analyze the following: (a) insulin secretion, (b) insulin sensitivity levels, and (c) the level of IL-17 in peripheral blood in nondiabetic FDRs, divided into subgroups based on the stage of prediabetes, and in patients with R-T1D.

## 2. Materials and Methods

### 2.1. Subjects

As described previously [15], we investigated 51 FDRs related to patients with T1D, 24 patients with R-T1D in the insulin-requiring state (IRS), 10 patients with R-T1D in clinical remission (CR), and 18 healthy controls (HC) who were not related to patients with T1D.

Inclusion criteria for FDRs were as follows: siblings and parents ≤45 years old were allocated into 2 subgroups: FDRs0 and FDRs1. In this context, FDRs0 included 34 FDRs with no glutamic acid decarboxylase (GAD) or tyrosine phosphatase insulinoma antigen-2 (IA-2A) antibodies in stage 0 with a lower risk for T1D development. As for the other group, FDRs1 comprised 17 FDRs with both autoantibodies in stage 1, with a higher risk for T1D development. They all had normal glucose tolerance, confirmed by a 2-h oral glucose tolerance test (2hOGTT). Only 2 subjects had positive GAD and IA-2A and impaired glucose tolerance (stage 2), and they were not included in the study.

We also included patients with R-T1D in stage 3, up to 3 months from the beginning of the overt disease, diagnosed according to the American Diabetes Association criteria [16], and in 2 different states during the disease: IRS and CR. IRS was defined as the necessity of insulin therapy to obtain normoglycemia [17]. Multiple daily insulin injections (human short-acting insulin before main meals + basal insulin at bedtime) were given. Patients in CR were at least 30 days without insulin therapy with optimal glycemic control [18]. The healthy controls had normal glucose tolerance, no detected GADA or IA-2A, and no family history of T1D.

Exclusion criteria were as follows: acute or chronic infectious, allergic, or autoimmune disease 6 months before inclusion, and use of immunomodulatory drugs or any other drug that could affect glucose tolerance 3 months before involvement in the study.

#### Research Design

The study protocol was previously described [15]. The metabolic investigations 2hOGTT, hyperinsulinemic-euglycemic insulin clamp, intravenous glucose tolerance test (IVGTT), glucagon stimulation test, and immunological investigations (detection of autoantibodies and cytokine levels) were performed at the Clinic for Endocrinology, Diabetes, and Metabolic Diseases, University Clinical Center of Serbia. All the subjects gave informed consent to be included, according to the Helsinki Declaration and the Institutional Review Board (Ethic Committee of the Faculty of Medicine, University of Belgrade, decision number 1600/I-21, 3 July 2006) approved the investigation.

All FDRs and controls underwent a 2-h OGTT to evaluate glucose tolerance status. Each subject underwent an IVGTT after a hyperinsulinemic-euglycemic clamp to evaluate insulin secretion and insulin sensitivity, respectively, and a glucagon stimulation test to detect endogenous insulin secretion capacity. The metabolic investigations were performed in euglycemia, without ketosis, at least 4 days apart from each test. In addition, we detected GADA and IA-2A and measured the level of IL-17 in all subjects.

### 2.2. Methods

#### 2.2.1. Detection of Glucose Tolerance Status

A 2-h OGTT with 75 g of glucose was performed by orally ingesting a 50% glucose solution for 3 min. After overnight fasting, venous blood samples were taken immediately before (0 min) and after glucose stimulation in the 120th minutes of the test for glycemia level [19]. The glycemia was analyzed with the enzymatic method on Cobass 6000 (Roche Cobas 6000 Chemistry Analyzer (Roche Diagnostics Corporation, Indianapolis, IN, USA)).

#### 2.2.2. Detection of Insulin Secretion Level

We tested insulin secretion with 2 well-validated stimuli, glucose and glucagon. The modified IVGTT was performed immediately after a 2-h euglycemic hyperinsulinemic clamp, while insulin infusion was continued with an infusing bolus of 0.3 g/kg in 50% glucose in half a minute [20]. Blood samples for plasma glucose and plasma insulin were drawn before and during 8 min after the intravenous glucose load. We analyzed the early phase of insulin secretion expressed as the first phase of insulin response (FPIR). FPIR was expressed as the total insulin levels in the 2nd and 4th minutes after glucose load [20]. Low FPIR for all age categories is <81 μU/mL [21]. Plasma insulin was determined by radioimmunoassay (RIA) (INEP, Belgrade, Serbia).

The glucagon stimulation test for detection of endogenous insulin secretion capacity was as follows: 1 mg of glucagon was administered intravenously, and blood was drawn at 0 and 6 min thereafter [19]. C-peptide levels were measured by radioimmunoassay, according to the manufacturer’s instructions [22].

#### 2.2.3. Detection of Insulin Sensitivity Level

The euglycemic hyperinsulinemic clamp was conducted by using insulin (40 mU/min^−1^/m^−2^) and glucose infusion simultaneously to achieve and maintain a steady state of target plasma glucose values at 5.0 mmol/L (±5%). The steady-state period was 40 min (80–120 min of clamp). Insulin sensitivity was expressed as an M value (calculated based on the amount of glucose infused during a steady-state period, averaged over the final 40 min of the 2 h clamp [20]. We used a standardized protocol for hyperinsulinemic-euglycemic clamps, with a coefficient of variability of glucose infusion rate equal to or less than 5% [20]. The hand used for blood sampling was warmed with a heating pad and retrogradely cannulated to “arterialize” the venous blood and minimize the effect of venous sampling on insulin sensitivity measurements.

#### 2.2.4. Detection of GADA and IA-2

According to the manufacturer’s instructions, GADA and IA2 were measured using the radioimmunoassay method (CIS Bio International, Gif Sur Yvette, France). The levels higher than 1 U/mL were considered positive [15].

#### 2.2.5. Measurement of IL-17 Level in Peripheral Blood

IL-17 (BioLegend, San Diego, CA, USA) levels were measured in plasma samples using commercially available enzyme-linked immunosorbent assays (ELISAs), per the manufacturer-provided protocol [23].

### 2.3. Statistics

Data are expressed as mean ± SD. Normal distribution was tested with a Kolmogorov–Smirnov test. A Kruskau–Wallis test was used to detect statistically significant differences among observed categories. Two-tailed *p*-values less than 0.05 were considered significant. Statistics were performed using the Statistical Package for the Social Sciences (SPSS) software (Advanced Statistics, version 26.0, Chicago, IL, USA).

## 3. Results

### 3.1. Clinical Characteristics

The baseline clinical characteristics of subjects involved in this study are shown in Table 1 and reported previously [15]. All subjects investigated were non-obese adults, and subject groups were similar regarding age and body mass index (BMI) (Table 1).

### 3.2. Analysis of Insulin Secretion Level

**Figure 1:**
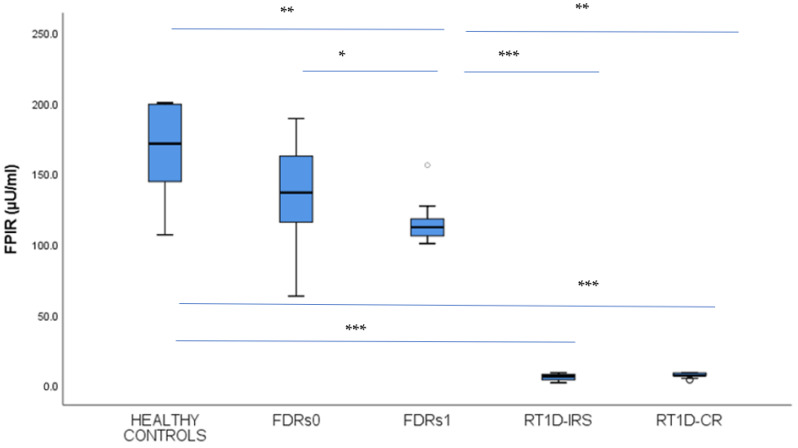
Insulin secretion expressed as the first phase of insulin response (FPIR). Comparison among first-degree relatives of T1D patients in stage 1 (FDRs1) and stage 0 (FDRs0) of T1D, recent-onset T1D patients (RT1D) in the insulin-requiring state (RT1D-IRS), clinical remission (RT1D-CR), and healthy controls (HC). The level of FPIR in peripheral blood was determined during the IVGTT. Results are expressed as mean +/− standard deviation. The horizontal lines show the median, and the box plots comprise the 25th and 75th percentiles and error bars of the 10th and 90th percentiles. Outliers are marked (white circles), and *p*-values < 0.05 *, <0.01 **, and <0.001 *** are indicated.

The levels of FPIR in FDRs1 were significantly lower compared to FDRs0 and healthy control subjects but still within the normal range for age (114.75 ± 13.02 vs. 138.71 ± 29.96 *p* < 0.05; 114.75 ± 13.02 vs. 167.83 ± 30.98 *p* = 0.001µU/mL). Moreover, FDRs0 and healthy controls did not differ significantly in FPIR (*p* = 0.07) (Figure 1). However, the level of FPIR in FDRs1 was significantly higher compared to patients with T1D in IRS and CR (114.75 ± 13.02 vs. 5.88 ± 2.32 *p* < 0.001; 114.75 ± 13.02 vs. 7.17 ± 1.73 *p* < 0.01 µU/mL) (Figure 1). At the same time, patients with T1D in IRS and CR had significantly lower FPIR levels compared to healthy controls and FDRs0 (*p* < 0.001) (Figure 1).

### 3.3. Defining the FPIR Level That Separates FDRs at Higher Risk for Developing T1D from Healthy Controls: ROC Curve Analysis of FPIR Scores

**Figure 2:**
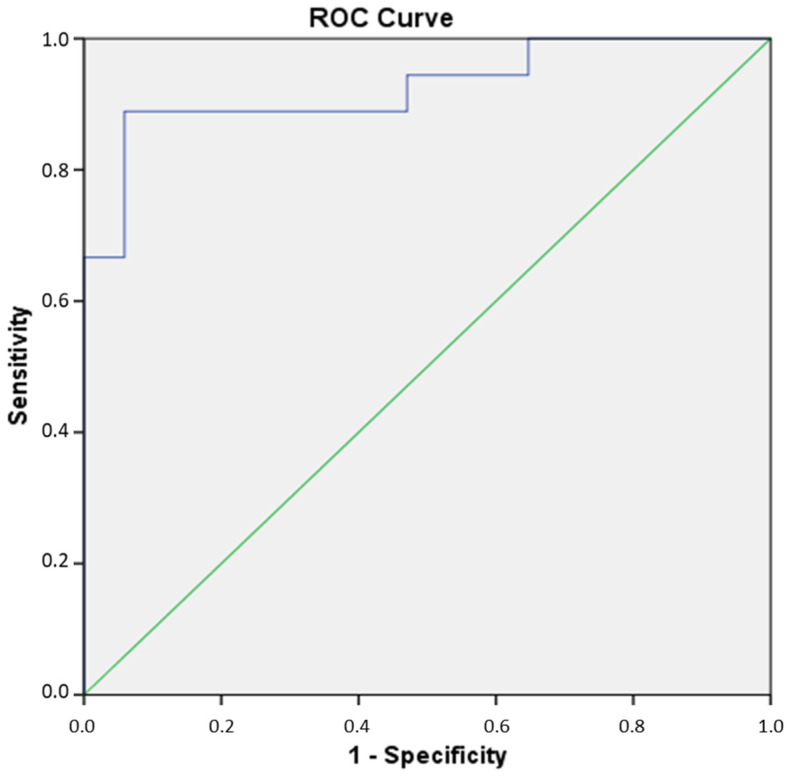
ROC curve analysis of FPIR scores. The blue line shows the AUC curve for FPIR, green line shows the reference line. AUC area: 0.925, *p* < 0.05.

We also determined the cut-off value, i.e., the level of FPIR at which it is possible to separate the FDRs1 from the healthy controls. Scrutinizing the FPIR scores, a binary logistic regression was conducted to define specificity and sensitivity cut-off points for predicting diagnostic categories: healthy control or FDRs1. We made a model showing an AUC value of 0.925 above the acclaimed threshold for excellent predicting results (0.9) (Figure 2).

**Figure 3:**
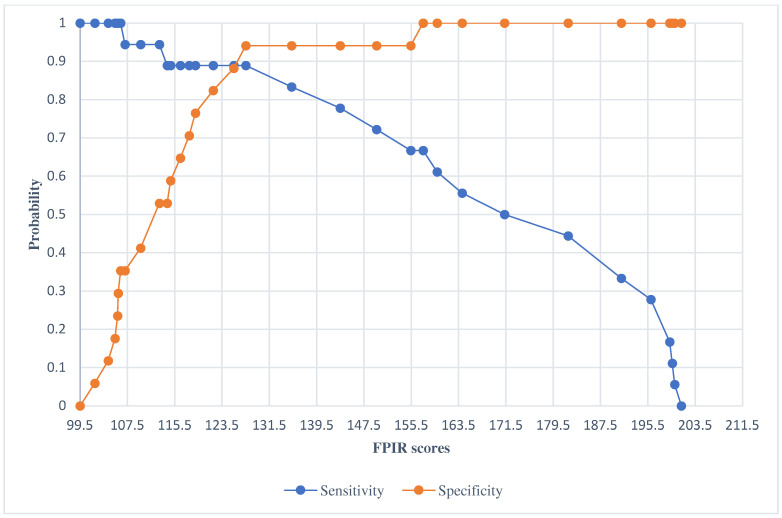
Overview of sensitivity and specificity analysis of FPIR scores for predicting diagnose category, with the cut-off value for FPIR = 125.76 µU/mL, and with the probability at 0.889.

Moreover, the model has been tested for its sensitivity and specificity values, where positive classes were associated with the prediction of FDRs1 (Figure 3). Taking into account the results accurately presented in Figure 2 and Figure 3, FPIR scores might be interpreted as predictors of diagnostic categories where the cut-off value for sensitivity and specificity is the value of 125.76 µU/mL (probability of 0.889). In this context, sensitivity or the probability to predict and identify subjects at higher risk for T1D development (FDRs1) is 88.9% if the FPIR level is lower than 125.76 µU/mL. Otherwise, specificity, or probability of falsely identifying subjects at risk (false positive FDRs1, i.e., healthy controls) rises significantly if FPIR levels are higher than 125.76 µU/mL.

### 3.4. Analysis of the Level of Endogenous Insulin Secretion

We demonstrated that in FDRs1, the level of basal and stimulated C-peptide was significantly lower in comparison to FDRs0 and healthy controls (basal C-pep.: 1.27 ± 0.11 vs. 1.45 ± 0.10 *p* < 0.01;1.27 ± 0.11 vs. 1.58 ± 0.13 *p* < 0.001; stimulated C-pep.: 1.91 ± 0.29 vs. 2.21 ± 0.23 *p* < 0.05; 1.91 ± 0.29 vs. 2.40 ± 0.29 nmol/l *p* = 0.001), although the values were in the reference range. At the same time, FDRs0 had similar basal and stimulated C-peptide levels as healthy controls (*p* = 0.058 and *p* = 0.069, respectively). On the other hand, FDRs1 had significantly higher levels of both basal and stimulated C-peptide than patients with T1D in IRS (basal C-pep.: 1.27 ± 0.11 vs. 0.16 ± 0.11 *p* < 0.001; stimulated C-pep.: 1.91 ± 0.29 vs. 0.35 ± 0.25 *p* < 0.001; nmol/l), while in CR, C-peptide levels were comparable (1.27 ± 0.11 vs. 0.57 ± 0.27; 1.91 ± 0.29 vs. 1.08 ± 0.48 nmol/l *p* = 0.13 and *p* = 0.084, respectively) (Table 2).

### 3.5. Analysis of Insulin Sensitivity Level

**Figure 4:**
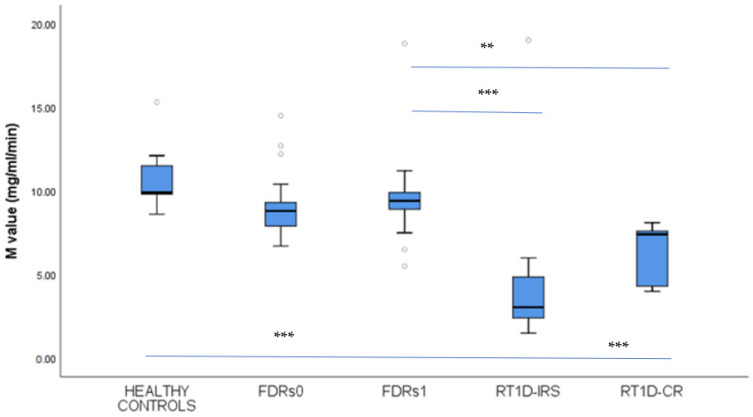
Insulin sensitivity expressed as M value: comparison among the first-degree relatives of T1D patients in stage 1 (FDRs1) and stage 0 (FDRs0) of T1D, recent-onset T1D patients (RT1D) in the insulin-requiring state (RT1D-IRS), patients in clinical remission (RT1D-CR), and healthy controls (HC). The level of M value was determined by the euglycemic hyperinsulinemic clamp method. Results are expressed as mean ± standard deviation. The horizontal lines show the median, and the box plots comprise the 25th and 75th percentiles and error bars of the 10th and 90th percentiles. Outliers are marked (white circles), and *p*-values < 0.01 ** and < 0.001 *** are indicated.

The level of M value in FDRs1 was not significantly different compared to FDRs0 and healthy controls, but it was significantly higher compared to T1D patients in IRS and CR (9.61 ± 2.76 vs. 10.47 ± 1.56; 9.03 ± 1.54 vs. 10.47 ± 1.56, *p* = 1.0; 9.61 ± 2.76 vs. 4.05 ± 3.46 *p* < 0.001; 9.61 ± 2.76 vs. 6.16 ± 1.83 *p* < 0.01 mg/mL/min) (Figure 4). At the same time, patients with T1D in IRS and CR had significantly lower M value levels compared to healthy controls (4.05 ± 3.46 vs. 10.47 ± 1.56 *p* < 0.001; 6.16 ± 1.83 vs. 10.47 ± 1.56 *p* < 0.001 mg/mL/min) (Figure 4).

### 3.6. Analysis of Proinflammatory Immune Response: IL-17 Levels in Peripheral Blood

**Figure 5:**
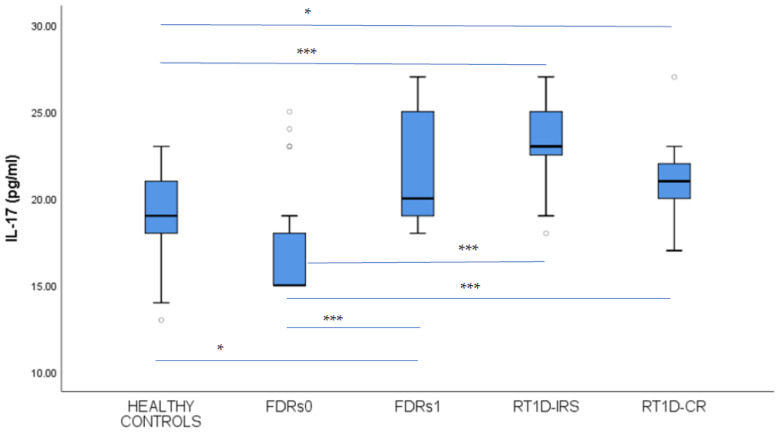
Interleukin 17 (IL-17) levels in peripheral blood: comparison among the first-degree relatives of T1D patients in stage 1 (FDRs1) and stage 0 (FDRs0) of T1D, recent-onset T1D patients (RT1D) in the insulin-requiring state (RT1D-IRS), patients in clinical remission (RT1D-CR), and healthy controls (HC). Results are expressed as mean ± standard deviation. The horizontal lines show the median, and the box plots comprise the 25th and 75th percentiles and error bars of the 10th and 90th percentiles. Outliers are marked (white circles), and *p*-values < 0.05 * and < 0.001 *** are indicated.

We found that FDRs1 had a significantly higher level of IL-17 compared to FDRs0 and healthy controls (21.76 ± 3.03 vs. 16.73 ± 2.88 pg/mL *p* < 0.001; 21.76 ± 3.03 vs. 18.83 ± 2.73 *p* < 0.05) (Figure 5), while FDRs0 had similar IL-17 levels to healthy controls (*p* = 0.059). Simultaneously, patients with T1D in IRS and CR had significantly higher levels of IL-17 than FDRs0 and healthy controls (23.42 ± 2.26 vs. 16.73 ± 2.88 *p* < 0.001; 21.33 ± 2.78 vs. 16.73 ± 2.88 *p* < 0.001 and 23.42 ± 2.26 vs. 18.83 ± 2.73 *p* < 0.001; 21.33 ± 2.78 vs. 18.83 ± 2.73 *p* < 0.05 pg/mL) (Figure 5). Additionally, IL-17 levels in IRS and CR did not differ from FDRs1, either between patients in IRS or in CR (*p* = 0.172, *p* = 0.763, *p* = 0.154, respectively).

## 4. Discussion

Our results have demonstrated that FDRs, adults in stage 1 with a higher risk for T1D development, had the diminished first phase of insulin secretion, together with impaired endogenous insulin secretion capacity but without changes in insulin sensitivity levels. In contrast, patients with R-T1D had a reduction in both insulin secretion and insulin sensitivity levels. Furthermore, in CR, the level of insulin sensitivity increased but without reaching the level in healthy controls. Additionally, FDRs1 with higher risk and patients with overt T1D in both states during the clinical course had profound proinflammatory immune responses. With this in mind, we consider that the risk and beginning of T1D might be potentially inflected on the level of IL-17.

Previous studies, performed mainly in children, suggested that defects in insulin secretion, high-risk HLA genotypes, age at onset, and the number and type of antibodies to β-cell antigens underlie the progression of T1D [24,25]. Considering that numerous studies performed IVGTTs, we wanted to better characterize insulin secretion during different stages in pre- and overt-T1D in adults. So, we have completed two complementary tests: an IVGTT with glucose and a glucagon stimulation test with glucagon as a stimulant for insulin secretion.

We found diminished FPIR in normoglycemic FDRs1, then in FDRs0 and healthy controls, although still in the normal range for adults. Similarly, FPIR as well as the other dynamic indices of insulin secretion, were lower in antibody-positive subjects than in subjects without autoantibodies, which is in line with our results [25,26]. On the other hand, previous studies reported markedly reduced FPIR in FDRs in advanced stage 2 prediabetes, as well as those with impaired glucose tolerance, higher titers of autoantibodies to β-cell antigens, and high predicted risk for overt T1D [10,27].

However, we determined the cut-off value 125.7 μU/mL as a valuable predictor of stage 1 FDRs and increased risk for T1D development, even though it is in the reference FPIR range. Therefore, according to our findings, FDRs1 at higher risk might be identified earlier, even when glycemia in OGTT and FPIR are still within the normal range.

Additionally, we confirmed the lowest FPIR later in the course of T1D, in RT1D. It is reported that β-cell glucose sensitivity, as the capability of β-cells to respond to fast glycemia fluctuations, drops first in progressors to overt T1D [28]. This early defect appears when there are no impairments of insulin secretion or insulin sensitivity. However, later, the loss of β-cell glucose sensitivity will increase glycemia, and even mild hyperglycemia due to glucose toxicity will cause further deterioration of FPIR and glucose tolerance to overt T1D, as in our research [28,29].

In addition, we detected diminished endogenous insulin secretion in FDRs1, then in FDRs0 and healthy controls, although once again the capacity was still within the normal range. The data about residual insulin secretion in different stages of prediabetes are limited. This study’s results indicated that the examined children, FDRs in stage 2 with impaired glucose tolerance, showed a decrease in basal C-peptide levels [30]. However, our FDRs had normal glucose tolerance but lower than normal levels of endogenous insulin secretion. A recently finished study demonstrated that performing hybrid closed-loop glucose control with an insulin pump in R-T1D in children did not stop the fall in endogenous insulin secretion [31]. It suggested that obtaining optimal glycemic control and eliminating glucose toxicity was not sufficient to decelerate the decline of endogenous insulin secretion powered by autoimmune destruction, which is in accordance with our findings.

However, it is suggested that in progressors, stimulated C-peptide levels decrease even more than two years before T1D onset [32], while in non-progressors they remain intact [33]. Similarly, it was shown that autoantibody-positive children progressing towards overt T1D have lower fasting and stimulated C-peptide levels than children with no autoantibodies [34]. Moreover, these changes are simultaneous with the early drop of FPIR in prediabetes [8]. At the same time, a decrease in the second phase of insulin response detected in the subjects in stage 1 might imply a defect in the translocation and maturation of insulin granules [35], which might underlie our findings, too.

On the other hand, in contrast to our results, the study which investigated adult FDRs detected a positive correlation between C-peptide and insulin autoantibodies, suggesting the compensatory rise of β-cell secretion [36]. Opposite to us, they included FDRs with one autoantibody and detected fasting C-peptide, only. Moreover, several studies suggested less efficient insulin processing and an increase in the proinsulin/C-peptide ratio in nondiabetic FDRs [37,38], but in the population of young children with prediabetes.

Finally, we found similar levels of insulin sensitivity among FDRs and healthy controls irrespective of stage, followed by a marked reduction in overt T1D. Our results are in line with published findings about similar levels of insulin sensitivity in autoantibody-positive and autoantibody-negative children [39]. It is important to emphasize that we used the golden standard to assess the insulin sensitivity level, the hyperinsulinemic-euglycemic clamp, which is a time-consuming and technically challenging procedure. At the same time, it was suggested that insulin insensitivity measured by the homeostatic model assessment index (HOMA-IR) may underestimate its actual level [40].

However, there is a positive correlation between insulin insensitivity and obesity, and in children with T1D, an increased BMI was found in the first year of life [41], while the prevalence and titer of GADA are associated with BMI in FDRs [42]. Despite those facts, BMI does not reflect visceral obesity, which affects insulin insensitivity, while body composition analysis was not performed [43,44]. Additionally, insulin insensitivity may reflect a more aggressive form of autoimmune disease, via immunoinflammatory factors that mediate both β-cell destruction and insensitivity [45,46]. In our study, the subjects were not obese and were matched according to BMI.

In contrast to our results, the association between stage 1 in children and pronounced insulin insensitivity was reported recently [35]. Moreover, progression to stages 2 and 3 in FDRs1 was accelerated with higher HOMA-IR but after adjustments for insulin secretion [47]. In our study, patients with overt T1D had a profound decrease in insulin secretion and sensitivity levels. It might be potentially mediated by decreased expression of growth hormone receptors in the liver, increased visceral adiposity, free fatty acids, and suppression of insulin receptor substrate-1 activity [48]. Later, in CR, after the elimination of glucose toxicity, insulin sensitivity is shown to rise but does not reach the level existing in the healthy controls.

Finally, bearing in mind that the decline in insulin secretion and modulation of insulin sensitivity is underlined with the autoimmune attack, we evaluated the proinflammatory immune response detecting IL-17 levels in the peripheral circulation. In that context, FDRs1 as well as patients with R-T1D had increased proinflammatory cytokine IL-17. Previously, it has been shown that IL-17A is a pro-inflammatory cytokine [49], stimulating the recruitment of other immune cells into insulitis and the progression of T1D [50], which might be the background for our findings and a potential mechanistic link, implicating the role of IL-17 in starting and promoting inflammatory attack in subjects at higher risk of T1D development. Furthermore, the data suggested increased levels of IL-17A in the peripheral blood of FDRs1 children correlated with increased destruction of β-cell mass and dysglycemia [51,52], which is partially in line with our investigation, especially when considering that our adult FDRs1 were euglycemic, but with diminished FPIR and endogenous insulin secretion.

We also detected increased IL-17 in overt R-T1D. In this context, there are investigations of children with overt T1D, in line with our research in adults, that demonstrated upregulation of Th17 immunity in the peripheral circulation and pancreatic lymph node [53,54,55]. Other studies showed that R-T1D subjects had up to 3-fold higher levels of CD4+ and CD8+ T cells that secrete IL-17 compared to controls [56]. Interestingly, long-term T1D subjects also showed a moderate increase in IL-17-secreting cells [57]. In contrast to our findings, similar levels of IL-17 in T1D patients and healthy controls were reported [58]. Furthermore, the study conducted on children with R-T1D surprisingly showed a positive correlation between Th17 and the incidence of CR [59]. Moreover, studies in animal models suggest that IL-17-secreting cells are a heterogeneous and modifiable cell population with a phenotype depending on immunologic and metabolic milieu [51], which might explain the contradictory findings.

Novel findings in children with R-T1D using ustekinumab, which blocks the development of Th17 cells, showed better endogenous insulin secretion and decreased autoantigen-specific IL-17A-secreting T cells [60]. Furthermore, a study with IL-17 blocking drug ixekizumab in RT1D patients is in progress, and our results additionally imply that these drugs might be used earlier in prediabetes, as in FDRs1. This drug might be involved in stopping the spreading and intensification of IL-17 potentiated proinflammatory response, which promotes the initial destruction of inflamed β-cells in subjects at risk for T1D development.

Finally, imatinib, a tyrosine kinase inhibitor, improved β-cell glucose and insulin sensitivity in R-T1D but surprisingly had no effects on proinflammatory immune responses [61,62]. Moreover, a recent paper pointed out the importance of earlier and more accurate detection of β-cell function loss in the earliest preclinical stage 1, bearing in mind that it is underestimated [63]. In this context, our findings might contribute as an attempt to better characterize the initial loss of β-cell mass/function and suggest the necessity of combined metabolic and immune intervention in the earliest stage of preclinical T1D. The potential protective effect of novel agent SGLT2 inhibitors on insulin secretion in pre-T1D remains to be investigated, especially in regard to conflicting results obtained from animal models [64]. Moreover, although GLP1-RAs achieve promising results only in animal models, potential implementation as an adjunct therapy in stage 3 T1D remains to be further clarified [65].

Our investigation’s limitations include a modest number of investigated subjects and a cross-sectional design, which cannot predict causality among parameters. Furthermore, we did not analyze relatives in stage 2, bearing in mind that the focus of our study was on early and not advanced and rarely detected stage 2 pre-T1D, although this would certainly complete the comparison of the analyzed metabolic and immunological parameters during the entire course of prediabetes. However, we investigated well-defined and homogeneous groups of adult subjects, using complementary and technically challenging methodology for metabolic testing. Importantly, most of the previous studies were performed in the pediatric population, where autoimmunity plays the main role in pathogenesis, and with differences compared to adults, starting from insulitis to clinical manifestation of T1D [66].

## 5. Conclusions

In conclusion, these results might imply that early changes in pre-T1D might involve an initial decline of insulin secretion associated with a pro-inflammatory attack, which does not influence insulin sensitivity, whereas later, insulin sensitivity deterioration seems to be associated with the prominent reduction in insulin secretion. Further studies are needed to include, more specifically, adult subjects exploring metabolic/immunological imprints in the early stage of preclinical T1D.

## Figures and Tables

**Table 1:** Baseline clinical characteristics of the study population. First-degree relatives of patients with T1D in stage 1 (FDRs1) and stage 0 (FDRs0), patients with recent-onset T1D (R-T1D) in the insulin-requiring state (IRS), patients in clinical remission of RT1D (RT1D-CR), and healthy controls (HC).

	FDRs1	FDRs0	RT1D-IRS	RT1D-CR	HC
Number	17	34	24	10	18
Gender (m/f)	4/13	18/16	15/9	4/6	2/16
Age (yrs.)	29.82 ± 8.83	26.44 ± 6.09	26.43 ± 6.02	26.22 ± 5.06	28.18 ± 7.21
BMI (kg/m^2^)	23.71 ± 2.66	22.69 ± 3.72	21.40 ± 3.47	22.12 ± 2.71	22.00 ± 4.21
Duration of T1D (months)	/	/	2.30 ± 0.52	9.20 ± 2.68	/
HbA1c (%)	/	/	9.70 ± 0.86	7.06 ± 0.42	/
FPG (mmol/L)	4.84 ± 1.06	4.22 ± 1.31	5.2 ± 0.8	5.8 ± 0.4	4.50 ± 1.20

Body mass index (BMI), HbA1c (glycated hemoglobin), FPG (fasting plasma glycemia).

**Table 2:** Descriptive analysis of endogenous insulin secretion capacity (C-peptide levels in glucagon stimulation test) in all subjects involved in the study (first-degree relatives of patients with T1D at stage 0 (FDRs0) and stage 1 (FDRs1) of T1D, patients with recent-onset T1D (R-T1D) in the insulin-requiring state (RT1D-IRS), patients in clinical remission of T1D (RT1D-CR), and healthy controls (HC)). Data are expressed as mean ± std. The Kolmogorov–Smirnov test was used to test normal distributions (*p*< 0.001). The Kruskal–Wallis (KW) test was performed; ***—*p* value < 0.001; **—*p* value < 0.01; *—*p* value < 0.05.

Indicators						KW
		FDRs0	HC	RT1D-IRS	RT1D-CR	
C-peptide 0 min (nmol/l)	1.27 ± 0.11	1.45 ± 0.10	1.58 ± 0.13	0.16 ± 0.11	0.57 ± 0.27	FDRs1 < HC *** FDRs1 < FDRs0 ** FDRs1 > IRS *** FDRs1 vs. CR NS FDRs0 vs. HC NS
C-peptide 6 min (nmol/l)	1.91 ± 0.29	2.21 ± 0.23	2.40 ± 0.29	0.35 ± 0.25	1.08 ± 0.48	FDRs1 < HC ** FDRs1 < FDRs0 * FDRs1 > IRS *** FDRs1 vs. CR NS FDRs0 vs. HC NS

## Data Availability

The data presented in this study are available on request from the corresponding author. The data are not publicly available, as they include sensitive clinical information.

## Abbreviations

The following abbreviations are used in this manuscript:

T1D	Type 1 diabetes
FDRs	First-degree relatives
IRS	Insulin-requiring state
CR	Clinical remission
IL-17	Interleukin 17
GAD	Glutamate acid decarboxylase antibodies
IA-2A	Tyrosine phosphatase insulinoma antigen-2 antibodies
SGLT2i	Sodium-glucose cotransporter inhibitors
GLP1-RA	Glucagon-like peptide receptor agonists
